# Application of CRISPR-Cas system in the diagnosis and therapy of ESKAPE infections

**DOI:** 10.3389/fcimb.2023.1223696

**Published:** 2023-08-17

**Authors:** Yizheng Qian, Dapeng Zhou, Min Li, Yongxiang Zhao, Huanhuan Liu, Li Yang, Zhiqin Ying, Guangtao Huang

**Affiliations:** ^1^ Department of Burns and Plastic Surgery, Affiliated Hospital of Zunyi Medical University, Zunyi, China; ^2^ The Collaborative Innovation Center of Tissue Damage Repair and Regeneration Medicine of Zunyi Medical University, Zunyi, China; ^3^ Department of Burn Plastic and Wound Repair Surgery, The Affiliated Hospital of Youjiang Medical University for Nationalities, Baise, China; ^4^ Department of Burn and Plastic Surgery, Department of Wound Repair, Shenzhen Institute of Translational Medicine, The First Affiliated Hospital of Shenzhen University, Shenzhen Second People’s Hospital, Shenzhen, China

**Keywords:** CRISPR-Cas, ESKAPE, pathogen, infection, diagnosis, therapy

## Abstract

Antimicrobial-resistant ESKAPE (*Enterococcus faecium*, *Staphylococcus aureus*, *Klebsiella pneumoniae*, *Acinetobacter baumannii*, *Pseudomonas aeruginosa*, and *Enterobacter* species) pathogens represent a global threat to human health. ESKAPE pathogens are the most common opportunistic pathogens in nosocomial infections, and a considerable number of their clinical isolates are not susceptible to conventional antimicrobial therapy. Therefore, innovative therapeutic strategies that can effectively deal with ESKAPE pathogens will bring huge social and economic benefits and ease the suffering of tens of thousands of patients. Among these strategies, CRISPR (clustered regularly interspaced short palindromic repeats) system has received extra attention due to its high specificity. Regrettably, there is currently no direct CRISPR-system-based anti-infective treatment. This paper reviews the applications of CRISPR-Cas system in the study of ESKAPE pathogens, aiming to provide directions for the research of ideal new drugs and provide a reference for solving a series of problems caused by multidrug-resistant bacteria (MDR) in the post-antibiotic era. However, most research is still far from clinical application.

## 1 Introduction

ESKAPE pathogen infection often leads to high mortality, and expensive treatment fee, which often brings a heavier financial burden. The U.S. Centers for Disease Control and Prevention (CDC) estimates that ESKAPE pathogens cause more than 2 million infections and at least 29,000 deaths annually in the United States (Sivalingam et al., 2019; De Oliveira et al., 2020; Sutradhar et al., 2021). At the same time, the cost of the five bacteria in the ESKAPE pathogens (*Staphylococcus aureus*, *Escherichia coli*, *Klebsiella pneumoniae*, *Acinetobacter baumannii*, and *Pseudomonas aeruginosa*) in the United States is approximately $2.9 billion per annum (Shrestha et al., 2018). For some middle- and low-income developing countries, the situation is a lot more severe. Global antibiotic consumption increased by 65% between 2000 and 2015, mainly inspired by low- and middle-income countries (Klein et al., 2018).

Mastering some genetic characteristics of ESKAPE pathogens is one of the important strategies to avoid this series of problems. *A. baumannii* can produce carbapenemase, which is a class of β-lactamases that can hydrolyze carbapenem antibiotics (Wu et al., 2020), while the drug resistance of *P. aeruginosa* is more complicated, which may be related to the production of the enzyme, membrane permeability and changes in target sites, biofilm synthesis, and the generation of adaptive resistance (Du et al., 2020). *Enterobacter* spp., multidrug-resistant *P. aeruginosa*, and multidrug-resistant *A. baumannii* have the ability to produce β-lactamase, which hydrolyzes β-lactam antibiotics (such as penicillins, cephalosporins, and carbapenems), thus making the drugs lose their antibacterial properties. Acquired resistance is more destructive, which means that bacteria acquire resistance-related genes by means of mobile elements (such as zygotic plasmids, transposons, insertion sequences, and integration) (Wieland et al., 2018). ESKAPE pathogenic bacteria have strong mutation ability and fast reproduction speed, and the adaptive gene mutations generated by them can spread rapidly through plasmids. Among them, clinical isolates of *K. pneumoniae* usually carry diverse conjugative plasmids (including fertility plasmid and resistant plasmid), allowing drug resistance to disseminate among strains or even between different strains through conjugation (Sun et al., 2019).

In addition, some tangible reasons play an important role. Among them, the irregular use of antibiotics has traditionally been part of the more recognized and difficult problems in the world. Improper selection of antibiotics, insufficient doses, and poor patient compliance with antibacterial therapy will increase antibiotic resistance. The reason may be that the academic community’s incomplete understanding of the mechanism of ESKAPE pathogens resistance and unbalanced economic factors restricts the progress of recent drug research and development. Being dependent on statistics, from 2007 to 2009, a total of 40 million antibiotic prescriptions were issued in outpatient clinics in the United States, of which 27 million (67.5%) were proved to be unnecessary (Shapiro et al., 2014).

**Figure 1:**
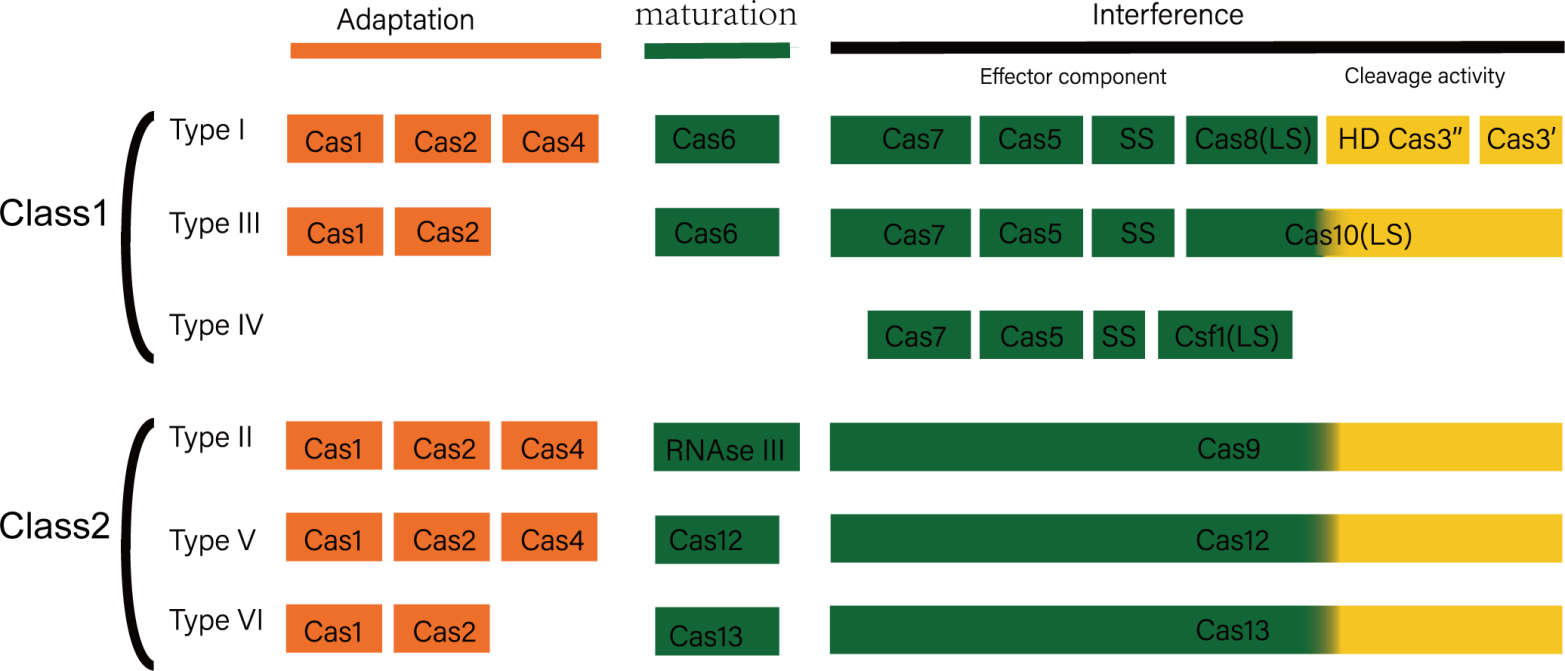
Composition and function of CRISPR-Cas system. CRISPR/Cas system is an adaptive immune defense formed by bacteria and archaea in the long-term evolution process, which fights against invading viruses and foreign DNA through three different but continuous stages: adaptation, crRNA maturation, and interference. CRISPR-Cas system mainly includes two categories: category 1 and category 2. Type 1 CRISPR-Cas systems include types I, III, and IV, and type 2 systems include types II, V, and VI. The first type of CRISPR-Cas system uses the cooperation of multiple Cas proteins to interfere, while the second type of system is interfered by a single protein.

Antibiotics research and development investment costs a fortune, along with the long development cycle, the high failure rate, and the low return on investment. On a global scale, each antibiotic takes an average of 11.8 years to develop at a cost of USD$ 1.5 billion (Towse et al., 2017). This has led to the reluctance of most pharmaceutical companies to develop antibiotics. As an oxazolidinone antibiotic that can effectively deal with ESKAPE pathogens, linezolid has limited its promotion due to the high production cost. In addition to the cost of product development, environmental pollution caused by the production of antibiotics has also become another obstacle on the road to antibacterial. Most of the pharmaceutical wastewater are antibiotic production wastewater, and the Dichromate Index (CODcr, that is, the chemical oxygen consumption measured in accordance with using potassium dichromate as an oxidant) in such wastewater is high, so are the biological toxicity and chromaticity. In addition to that, there are extremely fluctuating pH and complex governance processes (Qin et al., 2016). Traditional methods are time consuming, consumable, and inefficient to deal with drug-resistant bacterial infections. In recent years, some innovative strategies to deal with ESKAPE pathogens have emerged and have gradually grown up to become a hot spot in the field of clinical trials and antibacterial therapy. The scholarly community is trying to alleviate the pressure of nosocomial infections brought about by drug-resistant bacteria to some extent through these potential emerging antibacterial strategies (Gonzalez de Aledo et al., 2021). The CRISPR-Cas system, consisting of CRISPR sequences and CRISPR-associated proteins (Cas proteins), is an adaptive immune system restricted to bacteria and archaic, which can protect the host from the invasion of foreign nucleic acids. According to the composition and function of Cas proteins. CRISPR-Cas systems can be classified into two main classes, and each one divided into several types (**Figure 1**) (Strich and Chertow, 2019). At present, research on CRISPR-Cas system is mainly focused on the molecular mechanism of its immune function and its application in the field of gene editing. The research on the influence of CRISPR-Cas system on the stability of the host genome is quite rare. A related study found that among the 4,500 spacers derived from the NCBI database, 35% of the spacers had homology to the microbial genome, and some of these spacers targeted sequences on mobile genetic elements. The above results indicate that these genome-targeting spacers are not accidental, and they may play some important roles in the evolution of bacteria. Bacteria’s intrinsic CRISPR system allows bacteria to be immune to foreign DNA such as phage. The spacer sequence in the CRISPR structure plays an important part in the immune process. Research revealed that the spacer sequence has the phenomenon of insertion and selective deletion during bacterial evolution, which makes the CRISPR structure polymorphic, and there are differences between different strains of the same species. Therefore, the CRISPR structure can be employed as an ideal site for bacterial typing and evolutionary research (Mohanraju et al., 2016).

In addition, CRISPR is a gene-editing tool that can also be used for pathogen detection. It involves designing a specific CRISPR system that includes Cas proteins and guide RNA (gRNA) that matches the target pathogen’s DNA sequence. When the Cas protein binds to the target DNA, it can cut the DNA or bind to it, depending on the specific system used. In pathogen detection, Cas proteins are used to target the pathogen’s DNA. If the target DNA is present in a sample, the Cas protein will either cut it or bind to it, producing detectable signals such as specific DNA fragments or color changes. By detecting these signals, we can determine the presence of the target pathogen. For example, Janice’s team found that Lachnospiraceae bacterium ND2006 Cas12a (LbCas12a) has an extraordinary ability to non-specifically cleave ssDNA after binding to RNA-guided DNA (Janice S. Chen et al., 2018). Using this feature, combined with fluorescently labeled ssDNA signal reporter molecules, the DNA endonuclease-targeted CRISPR trans reporter (DETECTR) biosensing platform was developed, which can rapidly and accurately detect HPV and can distinguish HPV subtypes with very similar sequences. Combined with loop-mediated isothermal amplification (LAMP) technology, a highly sensitive detection of dispassionate human papillomavirus (HPV) was achieved. Utilizing various Cas12 effector proteins, researchers have developed a variety of new CRISPR/Cas12 biosensing platforms for pathogen detection, including HOLMES, HUDSON, and other CRISPR/Cas biosensing systems (Li et al., 2018). By combining RPA, LAMP, and other technologies, an ultra-detection of pathogenic bacteria has been achieved (Wu et al., 2017; Myhrvold et al., 2018; Li et al., 2019a). In order to shorten the detection time, Wang et al. attached Cas12a to the tube wall and added it to the system by centrifugation after RPA amplification to realize integrated detection, which could shorten the detection time to 30 min (Wang et al., 2019a). Finally, CRISPR can also be applied to the treatment of ESKAPE infections. Potential applications of CRISPR in ESKAPE treatment include gene editing to weaken drug resistance, gene regulation to control resistance-related gene expression, pathogen inactivation by cutting specific DNA sequences, and introducing antagonistic genes to combat drug-resistant bacteria. These applications show promise in developing new therapeutic strategies against ESKAPE infections, but further research and clinical studies are needed to validate their safety and effectiveness.

## 2 Application of CRISPR-Cas system in the diagnosis and therapy of ESKAPE infections

### 2.1 Application of CRISPR technology in the detection and diagnosis of ESKAPE infections

Most studies on pathogen detection using CRISPR have involved the following mechanisms. The CRISPR/Cas12 system is an RNA-guided DNA-targeting CRISPR system. Taking Cas12a as an example, the effector protein can catalyze the maturation of its own crRNA without the involvement of tracer RNA. The Cas12a–crRNA binary complex can achieve precise aiming at the target DNA sequence by targeting a PAM site rich in T nucleotides. CrRNA is usually composed of 19nt repeat sequence (repeat) and 23-25nt spacer sequence (spacer). The repeats contain palindrome sequences, which can form a stable stem-loop structure through base pairing, and its conformations stability is affected by the intramolecular interaction of hydrated magnesium ions (Mg(H2O)6)2+ and Cas12a. Cas12a recognizes and binds crRNAs being dependent on the length and sequence specificity of the stem-loop structure. Spacer sequences, which make up of RNA sequences transcribed from exogenous DNA, are bacterial antigens against the same invaders. After bacterial recognition of foreign DNA, Cas12a utilizes the RuvC domain of cis-cleave target DNA adjacent to the PAM sequence. After the dissociation and release of the DNA fragment at the distal end of the PAM, the cleavage activity of Cas12a trans-nuclease is activated, the signal reporter molecule can be cut in large quantities, and the specificity of the target nucleic acid can be achieved by detecting the corresponding signal (such as fluorescence) (Dong et al., 2016; Watters et al., 2018).

#### 2.1.1 Staphylococcus aureus

New advancements have also been made in the application of the CRISPR-Cas system for the detection of *S. aureus*. Zhou et al. developed a bacterial sensing strategy with the name of CCB detection (CRISPR-Cas13a based bacterial detection), taking advantages of CRISPR-Cas13a system, namely, the crRNA programmability and Cas13a “collateral effect” of promiscuous RNase activity upon target RNA recognition. *S. aureus* was selected as the model bacterium to validate the performance of CCB detection. The validation process involved four steps, namely, 1) straightforward extraction of genomic DNA; 2) specific gene amplification through PCR; 3) *in vitro* transcription; and 4) cleavage of reporter RNA, known as the “collateral effect,” to indicate the presence of the target analyte. Remarkably, CCB detection demonstrated successful detection of the target genomic DNA (gDNA) at concentrations as low as 100 aM. The limit of detection (LOD) was determined to be 1 CFU/mL, with a dynamic detection range spanning from 100 to 107 CFU/mL for *S. aureus*. The entire sample-to-answer process for this biosensor required <4 h. CCB detection exhibited excellent selectivity for *S. aureus*, with no interference from other bacterial species. Moreover, the application of CCB detection in real food samples, including those with both known and unknown levels of bacteria (whether spiked or non-spiked), yielded results comparable to the conventional culture-based counting method. Notably, CCB detection offered advantages such as reduced assay time and increased sensitivity. With its reliability, sensitivity, specificity, and simplicity, the proposed CCB-detection technique can be expanded and applied to the detection of other bacteria. It holds significant potential for a wide range of applications, including food safety inspections, disease diagnosis, and environmental monitoring (Zhou et al., 2020).

Meanwhile, Li et al. developed a new platform for MRSA (methicillin-resistant *S. aureus*) detection. Although the current diagnostic methods used in clinical practice, such as PCR and culture-based techniques, are commonly employed, they are not suitable for rapid point-of-care testing (POCT). With the recent advancements in CRISPR/Cas technology, new opportunities for rapid point-of-care detection have emerged. In the study undertaken by Yanan Li et al., a platform for the rapid, precise, and contamination-free detection of MRSA was developed by integrating the Cas12 system with recombinase polymerase amplification (RPA) in a single tube. By employing this approach, visual detection of MRSA could be achieved within a mere 20 min. The assay results, obtained using the one-tube RPA-CRISPR/Cas12a platform, can be visualized through lateral flow test strips (LFS) and fluorescence-based methods, including real-time and end-point fluorescence. This versatile platform allows for the specific detection of MRSA, with a sensitivity of 10 copies for the fluorescence method and a range of 10–100 copies for LFS. The results obtained from 23 samples of clinical MRSA isolates demonstrated a coincidence rate of 100% for the fluorescence method and 95.7% for LFS, as compared to qPCR. In conclusion, the one-tube RPA-CRISPR/Cas12a platform proves to be an efficient method for MRSA detection, holding significant potential for practical applications in future point-of-care testing (Li et al., 2022).

Su designed and synthesized specific primers based on the sequence of the conserved region of *S. aureus* thermostable nuclease gene (nyc) and established the isothermal amplification technology mediated by *S. aureus* recombinase by optimizing the reaction conditions, expressing and purifying CRISPR-Cas13a protein, designing specific crRNA, and using crRNA to guide CRISPR-Cas13a protein to detect RAA products; the sensitivity and specificity of the optimized method were evaluated, and this method and real-time PCR method were used to detect the golden yellow color in food samples. *Staphylococcus* was observed, and the consistency of the method was evaluated. The sensitivity of CRISPR-Cas13a-assisted RAA detection of *S. aureus* was 101 CFU/mL, which was higher than that of real-time PCR, approximately 102 CFU/mL; the detection time was only 30 min, and there was no cross-reaction with other food-borne pathogens. The positive rates of 80 food samples detected by this method and real-time PCR were both 8.75%, with high consistency (kappa=1, p>0.05). In the end, the conclusion is that the established CRISPR-Cas13a-assisted RAA method has the advantages of simplicity, rapidity, sensitivity, and specificity, and provides a modern technical means for the detection of *S. aureus* (Su et al., 2020).

#### 2.1.2 Klebsiella pneumoniae

*Klebsiella pneumoniae* is a prevalent culprit in hospital-acquired infections. An urgent need exists for a prompt, precise, and convenient detection approach to facilitate early diagnosis and targeted treatment of *K. pneumoniae* infections. To address this, Qiu et al. devised a novel assay called CRISPR-top (CRISPR-mediated testing in a single vessel). It combines the power of LAMP (loop-mediated isothermal amplification) with CRISPR/Cas12b-based detection, enabling a streamlined process performed at a constant temperature. Their optimized *K. pneumoniae* CRISPR-top assay accurately identifies the presence of *K. pneumoniae* strains within 60 min at a temperature of 56°C. By fine-tuning the reaction mixture composition, they achieved optimal results with 0.53 mM (each) FIP and BIP primers, 0.27 mM LF primer, 0.13 mM (each) F3 and B3 primers, and a 2 mM ssDNA fluorescence probe. Remarkably, their assay exhibits a detection limit of 1 pg genomic DNA per reaction, equivalent to 160 K*. pneumoniae* cells or 1.6 × 10^5^ CFU/mL in samples, surpassing the sensitivity of traditional LAMP methods by a factor of 10. Validation studies using a diverse panel of 105 strains, including *K. pneumoniae* clinical isolates and non-*K. pneumoniae* strains, demonstrated accurate identification rates. Additionally, they conducted a comprehensive evaluation of the *K. pneumoniae* CRISPR-top assay using 58 respiratory symptomatic sputum samples. Impressively, their assay achieved a specificity of 100% (33/33) and a sensitivity of 96% (24/25), yielding a positive predictive value of 100% (24/24) and a negative predictive value of 97.1% (33/34), surpassing the performance of conventional LAMP detection methods. In summary, the study conducted by Qiu and colleagues introduces the *K. pneumoniae* CRISPR-top assay as a rapid, straightforward, and highly specific means of detecting *K. pneumoniae* infections (Qiu et al., 2022).

#### 2.1.3 Acinetobacter baumannii

In 2020, Li developed a versatile CRISPR-Cas12a platform for detecting a wide range of analytes (Li et al., 2020), including *A. baumannii*, at ultralow concentrations. The platform utilizes LbaCas12a, which acts as a signal amplifier by recognizing single-stranded DNA intermediates generated by non-DNA targets. With the help of functional nucleotides, such as DNAzyme and aptamer, ultrasensitive bioassays for Pb2+ and *A. baumannii* were designed, achieving a limit of detection of approximately 0.053 nM and 3 CFU/mL, respectively. Additionally, the platform allows simultaneous detection of four microRNAs (miRNAs) without significant interference, indicating its potential for high-throughput analysis of miRNA expression profiles. Afterwards, in the year 2021, Wang developed a rapid platform that integrates multiplex PCR with CRISPR-Cas array for the detection of multidrug-resistant *A. baumannii* (MDRAB) (Wang et al., 2021). The platform utilizes a multiplex PCR amplification strategy to simultaneously amplify the housekeeping gene and four β-lactamase genes of *A. baumannii*. The platform also utilizes the indiscriminate single-stranded DNase activity of LbaCas12a to generate a single fluorescent signal for the multiplex PCR products. This platform enables genotypic antibiotic susceptibility testing (g-AST) of *A. baumannii* within 2 h, with a detection limit down to 50 CFU/mL. Their studies demonstrate the potential of the CRISPR-Cas12a method for rapid and specific detection of multiple genes, promoting its application in the diagnosis and treatment of multidrug-resistant bacteria.

#### 2.1.4 Pseudomonas aeruginosa

*Pseudomonas aeruginosa* is a significant bacterial pathogen responsible for hospital-acquired infections and poses a threat to patients with cystic fibrosis. In 2020, Mukama et al. introduced a novel approach called CIA (CRISPR/Cas and LAMP-based lateral flow biosensor) for the detection of *P. aeruginosa* (Mukama et al., 2020). By combining CRISPR/Cas systems (LbaCas12a and AaCas12b) with loop-mediated isothermal amplification (LAMP), the method achieves high sensitivity and specificity. It utilizes a lateral flow biosensor (LFB) and collateral cleavage of a biotinylated DNA reporter to detect the target gene. The CIA-based LFB demonstrates the ability to accurately distinguish *P. aeruginosa* from other bacteria in complex samples. Their cost-effective and efficient method shows great potential for clinical diagnosis of infectious diseases. Finally, Qiu et al. introduced another novel detection method called CRISPR-top assay (Qiu et al., 2023, which is based on CRISPR-Cas12b and allows the rapid and accurate identification of *P. aeruginosa*. The assay is performed within a single tube, requiring only one fluid-handling step and no specialized instruments. Under optimized conditions, including a temperature of 55°C and specific primer concentrations, the assay demonstrated high specificity, inclusivity, and exclusivity. With a limit of detection of 10 copies per reaction, the CRISPR-top assay exhibited promising results in the analysis of 46 respiratory specimens, showing a sensitivity of 85.3% and a specificity of 100%. Overall, their study suggests that the *P. aeruginosa* CRISPR-top assay can serve as an efficient and practical tool for rapid detection, particularly in resource-limited settings.

#### 2.1.5 Enterobacter species

A total of 135 strains with complete sequence and 203 strains with whole genome shotgun sequence of *E. coli* were identified in GenBank by Liang Wenjuan et al. using BLAST. Additionally, 361 strains of *E. coli*, including 38 strains of *E. coli* O157:H7, were identified in the laboratory using PCR. The identified strains were then analyzed using the CRISPR Finder tool. To compare the spacers, DANMAN was employed, and phylogenetic trees of the cas gene were constructed using Clustal X and Mega 5.1.Then, they obtained the following results. A new descriptive method was developed to investigate the positioning of CRISPR/cas in *E. coli*. The presence of CRISPR1 was detected in 77.04%, 100.00%, and 75.62% of the 135 strains with complete sequence, 203 strains with whole genome shotgun sequence, and 361 laboratory strains, respectively. Similarly, CRISPR2 was found in 74.81%, 100.00%, and 92.24% of the respective strains. However, CRISPR3 and CRISPR4 were only detected in 11.85%, 0%, and 1.39% of the mentioned strains. Among the strains analyzed, one strain from GenBank and two laboratory strains contained four CRISPR loci. Additionally, one *E. coli* strain in our dataset exhibited an insertion sequence downstream of the non-cas CRISPR1. A unique CRISPR was identified in eight strains of O55:H7, 180 strains of O157:H7, eight strains of O157: HNM, 40 strains of O104:H4, four strains of O145:H28, and across all 699 *E. coli* strains. The phylogenetic tree revealed two distinct groups based on the cas type, either I-E or I-F. The authors thus concluded that CRISPR/Cas has the potential to serve as a valuable molecular biomarker in epidemiological surveillance studies, enabling the identification of highly virulent or novel strains of *E. coli* (Liang et al., 2016).

### 2.2 Application of CRISPR-Cas system in the therapy of ESKAPE infections

**Figure 2:**
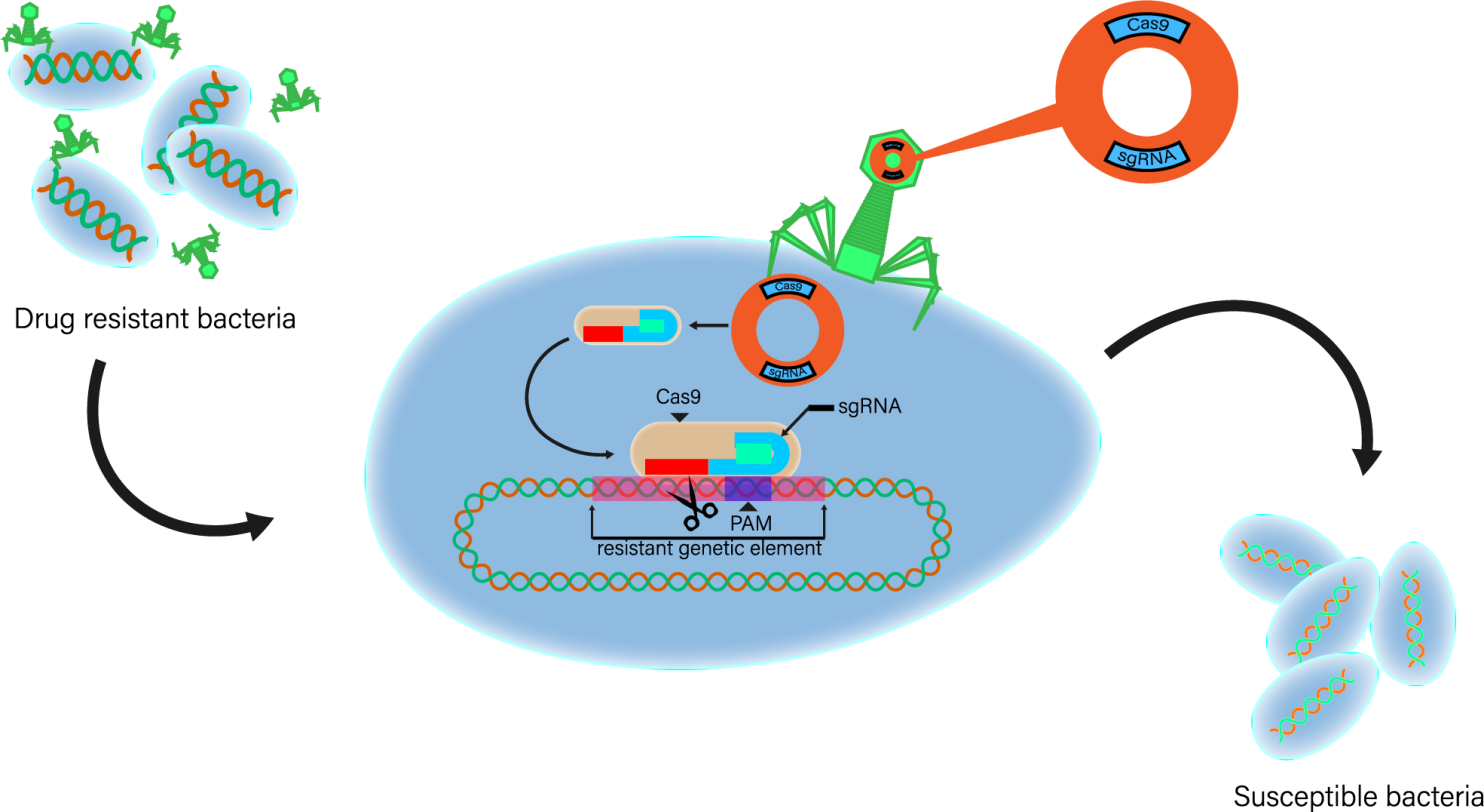
The second type of system is interfered by a single protein. Basic strategy of using exogenous CRISPR-Cas as antibacterial agent. CRISPR-Cas phage vector is a plasmid construct cloned with CRISPR-Cas element and phage packaging sequence. Phage carrier particles are adsorbed on bacteria, and plasmids carrying genes related to CRISPR-Cas system are transferred to drug-resistant bacteria. Exogenous Cas gene and sgRNA expression sequence were expressed in host cells. The cleavage of targeted drug-resistant genes by CRISPR-Cas system will lead to the loss of drug-resistant plasmids or DNA double-strand breaks. When bacteria cannot re-cyclize plasmids or repair their genomes, drug-resistant bacteria will die. Phage continues to enter the replication cycle and synthesize offspring and lyse cells, further optimizing the therapeutic effect.

Although CRISPR-Cas technology has shown its great efficiency in gene editing, it is hitherto not considered as a possible antimicrobial treatment because of the delivery issue. Most studies introduce the CRISPR-Cas system into their experimental bacterial cells using plasmid electroporation; however, this method apparently cannot be successfully performed *in vivo*. Thus, another method is to be considered such as phage delivery (Fage et al., 2021). As an accurate gene editing technology, CRISPR-Cas system can accurately edit the target gene at a fixed point. Based on this effect and combined with phage delivery, CRISPR-Cas system is theoretically enabled to exert an antimicrobial effect *in vivo*. The basic principle is that after infecting drug-resistant bacteria with phage carrying genes related to CRISPR-Cas system, the cleavage of drug-resistant genes by CRISPR-Cas system targeting bacteria will lead to the loss of drug resistance, and finally, the drug-resistant bacteria will recover their sensitivity to antibiotics (**Figure 2**) (Bikard et al., 2012; Bikard et al., 2014; Citorik et al., 2014; Fage et al., 2021). Direct use of CRISPR-Cas system can eliminate bacteria by targeting drug-resistant genes or virulence genes and at the same time limit the transfer and prevalence of harmful genes among microorganisms, which provides a new direction for the study of preventing and treating bacterial multidrug resistance at the gene level.

#### 2.2.1 *Enterococcus faecium* and *Enterococcus faecalis*

*Enterococcus* spp., a group of cocci that stain Gram-positive and typically arrange themselves in pairs or short chains, represents a genus of bacteria. While they are commonly found in the gastrointestinal microbiota, two specific species, namely, *E. faecalis* and *E. faecium*, are known to frequently cause infections. Their remarkable ability to endure on inert surfaces for extended periods has elevated their significance in the context of hospital-acquired infections. Mutations and/or excessive production of a penicillin-binding protein (PBP) called PBP5, belonging to the class B category, endow these bacteria with inherent resistance to a wide range of β-lactam antibiotics. However, it is noteworthy that ampicillin remains effective against the majority of *E. faecalis* strains. Moreover, enterococci possess intrinsic resistance to aminoglycosides, thereby limiting the available therapeutic options.

The horizontal transfer of antibiotic resistance genes limits the treatment options of infection (Liu et al., 2013). Price and his colleagues used *E. faecalis* T11 as a model organism to evaluate the role of CRISPR-Cas system in genome defense against common conjugated plasmids (Price et al., 2016). They proved that CRISPR-Cas, together with restriction modification, reduced plasmid acquisition by bacteria in biofilm by four times. This study highlights the importance of CRISPR-cas system of *E. faecalis* in regulating horizontal gene transfer. Understanding the basic biology and molecular basis of pathogenicity of *E. faecalis* is helpful to effectively improve and improve the ability to formulate treatment strategies. A basic part of the biological research of *E. faecium* depends on the ability to produce mutants, but this process is often time consuming and ineffective (Zhang et al., 2012). Maat described a method based on CRISPR-Cas9 to improve the current genetic toolbox of *E. faecium* (de Maat et al., 2019). In short, it is a mutant that uses CRISPR-Cas9 as an anti-selection strategy, relies on the high internal recombination rate of *E. faecalis* for allele exchange, and combines CRISPR-Cas9 to produce specific gene mutations in the chromosome of *E. faecium* more effectively.

So far, there have been almost no direct research results on using CRISPR-Cas for the treatment of *E. faecium*. In 2019, Rodrigues and colleagues presented a potential approach for treating *E. faecalis* using the CRISPR-Cas system (Rodrigues et al., 2019). They conducted a study where they successfully integrated a CRISPR system targeting the tetM and ermB genes, known for conferring resistance to tetracycline and erythromycin, respectively, into a pheromone-responsive plasmid (PRP) called pKH88[sp-tetM] and pKH88[sp-ermB]. The transmission of these plasmids was achieved through conjugation, using *E. faecalis* CK135 as the donor strain and *E. faecalis* OG1SSp as the recipient strain. *In vitro* experiments demonstrated the effective elimination of antibiotic resistance. Subsequently, using an *in vivo* C57BL6/J mouse model, the researchers observed that although the recombination rate was low, the transconjugants that successfully acquired the PRPs were unable to acquire erythromycin-resistance genes. This finding led the authors to propose the potential utilization of probiotic *E. faecalis* strains carrying these PRPs to impede the colonization of patients by resistant *E. faecalis* strains.

#### 2.2.2 Staphylococcus aureus

*Staphylococcus aureus* is a pathogenic bacterium that can provoke a variety of infectious diseases, which seriously threaten human life and health. It has been reported that antibiotic-resistant strains were found on the surface of public devices such as ATM keyboards (Akinola et al., 2022). Clinical overuse of antibiotics to treat *S. aureus* infections have led to the prevalence of drug-resistant *S. aureus*. Methicillin-resistant *S. aureus* (MRSA) is a class of *S. aureus* resistant to β-lactam antibiotics (Souza-Silva et al., 2022). The global spread of MRSA causes severe persistent infection. Methicillin resistance in MRSA is taken from the resistance gene mecA on the movable genetic element SCCmec, which encodes the low-affinity penicillin-binding protein PBP2a. SCCmec elements can spread in *Staphylococcus* by horizontal transfer, resulting in epidemics of drug resistance.

Guan mainly considered the effects of type III-A CRISPR-Cas system self-targeted attack on the resistance gene mecA on the movable genetic element SCCmec in MRSA clinical strains on host genome stability and drug resistance. Through analyzing the genome sequence of transformants, it was found that the CRISPR-Cas system targeting the genome is under a lethal effect on the host, but a small number of bacteria can escape the attack of CRISPR-Cas through genome remodeling. Most of the escapers are under a large-scale loss of genome sequence, which is a DNA fragment of approximately 16 kb including the target gene within the SCCmec. The deletion of the anti-mecA spacers or loss-of-function mutation in Cas genes required for targeting allowed the remaining escapers surviving. The transcription efficiency of different length leader sequences was analyzed by RT-qPCR, the result indicating that the effective length of the naive leader sequence in the AHI strain was 252 bp. Thus, the 252-bp leader was selected as the promoter for the construction of the CRISPR plasmid. Studies have shown that the targeted attack of the type III-A CRISPR-Cas system is transcription dependent, and the attack effect can only be exerted when the spacer targets the coding strand of the target. By changing the length of the spacer sequence, it was found that the length of the spacer did not affect the processing of crRNA, and the length of the mature crRNA was fixed, both 43 or 37 nt. However, the length of the spacer does influence the targeted attack efficiency of the CRISPR-Cas system. This research not only shows that the targeted attack of the CRISPR-Cas system on the genome can promote the remodeling of the genome but also further elucidates the molecular mechanism of action of the type III-A CRISPR-Cas system (Guan, 2019).

In the reported strains and the five newly discovered MRSA strains, the CRISPR-Cas system is bordering on SCCmec, which suggests that the integration site of the CRISPR-Cas system may be near the insertion position of SCCmec in the genome. Analysis of CRISPR sequences found that the repeats of the -a system in *Staphylococcus* were highly conserved, while most of the homologous sequences of spacers were consulted on the template strand of the cleavage region in phage. Furthermore, a comparison of six strains found that the AH1 strain with fewer spacers contained more resistance and virulence genes than the other five strains. They found that the target recognition is dependent on sequence complementarity between crRNA and target RNA and that Cas6 and CSM complexes are necessary for immune function. For functions other than immunity, they found that CRISPR can be associated with governing the expression of virulence-related genes in *S. aureus*, but the mechanism still needs further research to determine. An author supposed that these findings may provide an innovative insight into the application of CRISPR-Cas systems in the therapy of MRSA infection (Cao, 2017).

Yang used the sgRNAcas9 v2.0 software to design the oligos sequence targeting the mecA gene and lighted it with the vector pCas9, which was digested with endonuclease and picked up by gel, and successfully constructed the pCas9:mecA-C-terminal plasmid(Yang, 2016). The research results show that, first, among the 32 *S. araneus* strains with 45 confirmed CRISPR loci involved in the database, 19 strains (59.4%) all contained one confirmed CRISPR locus, and 40.6% of the strains’ genomes contained two confirmed CRISPR loci. The 45 repeat sequences included in the study can form a conservative dumbbell-shaped RNA secondary structure and can be split into 3 and 15 groups according to sequence similarity. In group 1, the RNA secondary structure with 5 base pairs in the stem is under a lower minimum free energy (MFE) than the structure with 3 base pairs. In group 2, the secondary structure with the longer stem holds the smallest MFE. In group 3, the number of base pairs in the stem is the same, and the resultant structure with superior “GC” content in the stem has a smaller MFE. Second, a total of five spacer sequences in strains 08BA02176 and MSHR1132 were like exogenous plasmid or phage DNA sequences, and one of the spacer sequences was completely consistent with a sequence in the plasmid encoding Panton–Valentine leukocidin. Third, CRISPR sites are separated into 17 types and are classified into three major groups. In group 1, the vast majority (75.0%) of the strains with the same CRISPR type also had the same MLST type. In the second group, the two CRISPR A strains were both MLST398. In group 3, strains with different CRISPR types correspond to different MLST types. Among the six protein-coding sites adjacent to the CRISPR site, more than three are similar in sequence to the protein-coding sites at the same position around other CRISPR sites, and most of the CRISPR sites adjacent to these similar protein-coding sites have the same CRISPR type (Kumari et al., 2022). Fourth, pCas9:mecA could successfully eliminate pET-21a(+)-mecA in *E. coli* BL21(D3) or significantly reduce its replication. The control plasmid pCas9 had no such effect. Neither pCas9:mecA nor the control PCas9 had obvious elimination effect on the pET-21a(+) control plasmid. It can be observed that the more bases in the stem of the resultant structure of the repeat RNA, the higher the “GC” content, the greater the possibility that CRISPR can function. The mecA plasmid can specifically target the mecA gene, thereby causing the elimination of the drug-resistant plasmid pET-21a(+)-mecA or greatly reducing its replication (to the extent that it is not visible by gel electrophoresis) to limit the drug-resistant gene mecA spread between strains, thereby immunizing susceptible strains (Folliero et al., 2022).

Jiang used CRISPR/Cas9 technology to construct srtA gene deletion strains and complemented strains of methicillin-resistant *S. aureus* USA300 and tested their effects on the virulence of the strains. Jiang designed three pairs of srtA gene sgRNAs and constructed pCasSA-sgRNA plasmids with pCasSA as the vector. After being modified by the defective *S. aureus* RN4220 strain, it was transferred into USA300, and the cleavage efficiency of pCasSA-sgRNA plasmid was tested (Jiang et al., 2020). The left and right homology arms of the srtA gene were amplified and fused and inserted into the pCasSA-sgRNA plasmid to construct the knockout plasmid pCasSA-sgRNA-srtA. The knockout plasmid was modified and transferred into USA300, and the srtA gene deletion strain was obtained by screening and identification. Jiang compared and assessed the differences in the growth of the USA300 strain, the survival rate of mice, the microbial load in organs, and the histopathological changes in the kidneys after the deletion of the srtA gene. At the same time, Jiang used the pLI50 plasmid as a vector to construct a complementing plasmid pLI50-srtA and a complementing strain to verify whether there are different in the phenotype of distinct strains. After chloramphenicol screening, Jiang obtained two gene deletion strains. Report to the wild-type strain, the deletion of the srtA gene did not affect the growth of the USA300 strain but significantly reduced the mortality of infected mice, the bacterial load of the heart and kidney, and the degree of renal tissue lesions. Its function was returned after complementation by the srtA gene. The gene editing method of methicillin-resistant *S. aureus* USA300 strain with srtA gene deletion and complementation was satisfactorily established.

Zhang used bioinformatics methods to examine the distribution of CRISPR, repeat sequences, spacer sequences, cps genes, and the relationship between the presence of CRISPR and the bacterial mecA gene (Zhang et al., 2019). The results showed that 196 confirmed CRISPR structures and 1,757 suspected CRISPR structures were found in the genomes of 325 *Staphylococcus* strains; 25 strains contained gas gene clusters, which could be divided into III-A (48.1%) and II-C (51.9%) %) two types; it is determined that most of the repeat sequences in the CRISPR array are 2, and there are 14 repeat sequences that can form a stem-loop structure. Chromosomal evolution analysis shows that they can be roughly classified into three categories. It was determined that 53 of the 437 spacer sequences in the CRISPR array matched plasmids or phages, and some sequences matched multiple plasmids or phages at the same time. The carrying rates of mecA in the presence and absence of gas gene clusters were 28.00% and 54.15%, respectively, and the difference was statistically significant (χ2 = 6.37, p<0.05), and the two were negatively correlated. From this, it can be concluded that the CRISPR-Cas system carrying rate in the *Staphylococcus* genome is low, and the structure and function of the locus and sans gene are not perfect. Only a few strains contain a complete CRISPR-Cas system. There are more suspicious structures, and the number of DRs and spacers is lower than that of other bacteria. An intact staphylococcal CRISPR-Cas system may limit horizontal transfer of the mecA gene.

#### 2.2.3 Klebsiella pneumoniae

*Klebsiella pneumoniae*, a facultative anaerobe, encapsulated, and Gram-negative rod, belongs to the Enterobacterales order and is commonly found in the gastrointestinal tract of humans. In its wild-type form, this bacterium exhibits intrinsic resistance to aminopenicillins (such as ampicillin and amoxicillin) and carboxypenicillins (like ticarcillin and piperacillin) due to the presence of the chromosomal β-lactamase SHV-1. However, the worrisome aspect lies in the ability of *K. pneumoniae* to acquire resistance to nearly all approved antimicrobials. This resistance is achieved through a combination of plasmid-mediated carbapenemases and other resistance mechanisms.

Yao’s research (Yao et al., 2022) focuses on the treatment potential of Carbapenem-resistant *K. pneumoniae* (CRKP). CRKP is a significant health threat due to its resistance to multiple drugs. The resistance in CRKP is primarily attributed to large plasmids containing multiple resistance genes. Understanding the function of these genes is crucial for combating CRKP infections. However, there is a lack of efficient genetic manipulation tools for studying plasmid-borne genes in clinical *K. pneumoniae*. Traditional gene knockout methods are time consuming and laborious, prompting researchers to explore the use of CRISPR systems. While CRISPR-Cas9 has been successful in deleting chromosomal genes in *K. pneumoniae*, it resulted in plasmid loss when targeting plasmid-borne genes. The low homologous recombination efficiency further hindered gene manipulation. To address these challenges, Yao’s research introduces a CRISPR interference (CRISPRi) system that utilizes a catalytically inactive Cas9 (dCas9) nuclease and a single-guide RNA (sgRNA). The CRISPRi system allows for the inhibition of gene expression without causing double-stranded breaks or relying on homology-directed repair. It has been widely used to study gene function in various organisms. The study establishes an all-in-one CRISPRi tool specifically designed for CRKP. This system effectively shuts down the expression of individual and multiple resistance genes on large multidrug-resistant plasmids. It also facilitates the exploration of potential operons. The CRISPRi tool enables the easy manipulation of multicopy genes on plasmids in clinically pathogenic bacteria, including *K. pneumoniae* and *E. coli*. In general, their research presents a new CRISPRi tool that offers a rapid and efficient approach for investigating the function of plasmid-borne genes in complex clinical isolates, particularly in the context of Carbapenem-resistant *K. pneumoniae*.

#### 2.2.4 Acinetobacter baumannii

*Acinetobacter baumannii*, a Gram-negative, belongs to the family Moraxellaceae in the class Proeo-bacteria of Eubacteria (Eze et al., 2018). *Acinetobacter baumannii* is currently one of the leading pathogens that cause nosocomial infections, including ventilator-associated and bloodstream infections (Harding et al., 2018). Feng utilized PCR product cloning and sequencing and CRISPR system bioinformatics analysis on 89 clinical isolates to study the regulation of the *A. baumannii* CRISPR system on target genes (Feng et al., 2019). The results showed that the two isolate (AB43 and ATCC19606) possessed the CRISPR system, and the genes targeted by their inter-regional regions included type IV secretory protein Virb5, Zona toxin protein, phage protein, and DNA polymerase. The CRISPR system inhibits the coding and expression of Virb5 and zona toxin protein genes, which may have a certain regulatory effect on the biofilm formation of *A. baumannii*.

In response to the problem that “although phage therapy can solve the drug resistance of bacteria, the specific recognition of phage and host bacteria limits its application,” He tried to use CRISPR-Cas9 technology to perform gene editing on the filament protein sequence of the phage genome. Therefore, the scope of its bactericidal spectrum can be broadened, the modification of the binding specificity of phage host can be realized, or it can provide certain ideas and operational paths for clinical treatment.

Wang and colleagues developed a CRISPR-Cas9-based, rapid genomic editing platform to analyze the mechanisms involved in oxidative stress in *A. baumannii* by introducing deletions, insertions, and point mutations. The platform works by coupling a Cas9 nuclease-mediated genome cleavage system with the RecAb recombination system. The authors also developed a cytidine base-editing system to allow programmed C to T conversions. Then, they took the advantages of these techniques to comprehensively analyze the possible resistance genes in a clinically isolated multidrug-resistant strain by generating premature stop codons close to the genes to unravel each of their distinct effect on drug resistance (Wang et al., 2019b). The research may provide certain guidance for clinical antibiotic selection.

#### 2.2.5 Pseudomonas aeruginosa

*Pseudomonas aeruginosa* is a versatile Gram-negative bacterium widely known for its opportunistic pathogenicity. It is commonly found in diverse environments, including soil, water, and hospital settings. With its impressive metabolic capabilities, *P. aeruginosa* can utilize a wide range of organic compounds as energy sources. This bacterium poses a significant threat in healthcare settings, particularly among immunocompromised individuals and patients with cystic fibrosis. *Pseudomonas aeruginosa* exhibits intrinsic resistance to many antibiotics and can acquire additional resistance mechanisms through genetic adaptations. Its ability to form biofilms further contributes to its persistence and chronic infections.

In 2020, Xiang’ study (Xiang et al., 2020) employs the CRISPR interference (CRISPRi) technology using a catalytically deactivated Cas9 (dCas9) to study gene regulation in *P. aeruginosa*. Unlike the wild-type Cas9, dCas9 does not cut DNA but can bind to target sites using guide RNAs (gRNAs) and inhibit the transcription of target genes. This approach allows for interference with the expression or function of essential genes without completely eliminating them. In this research, a CRISPRi platform was constructed for *P. aeruginosa* using an arabinose-inducible pBAD vector. The expression of the prtR gene, which cannot be knocked out by conventional methods, was downregulated using this system. prtR and prtN form a regulatory cascade controlling the production of pyocins, bacteriocins that mediate bacterial cell lysis. The PrtR protein inhibits the expression of pyocin genes by binding to the promoter region. By controlling gene expression in an arabinose concentration-dependent manner, the study demonstrated the suppression of pyocin expression, leading to bacterial cell death. RNAseq analysis of the prtR-knockdown strain provided insights into downstream genes regulated by prtR, including both known and previously unknown targets. The results highlight the valuable application of CRISPRi as a tool for studying essential genes in *P. aeruginosa*.

#### 2.2.6 *Enterobacter* species

*Enterobacter* spp. comprises a diverse group of Gram-negative bacteria that are widely distributed in various ecological niches. They belong to the family Enterobacteriaceae and are characterized by their rod-shaped morphology. *Enterobacter* spp. includes several clinically relevant species, such as *Enterobacter cloacae* and *Enterobacter aerogenes*. These bacteria are opportunistic pathogens and can cause a range of infections, including urinary tract infections, respiratory tract infections, and bloodstream infections, particularly in hospitalized patients and those with compromised immune systems. One of the concerning aspects of *Enterobacter* spp. is their ability to develop resistance to multiple antibiotics, including extended-spectrum β-lactams and carbapenems. This acquired resistance poses a significant challenge in clinical settings and underscores the importance of effective infection control measures and appropriate antibiotic stewardship.

Li utilized the disk diffusion method to detect its drug sensitivity and the PCR method to detect and compare the phylogenetic groups, drug resistance genes, and CRISPR system of *E. coli* cutoff from fecal samples of diarrhea patients and healthy people (Li et al., 2021). A total of 142 strains of *E. coli* were collected from 63 chronic diarrhea patients (disease group) and 79 healthy individuals (healthy group). The drug susceptibility results showed that the ampicillin resistance rate was 48.0%, and other antimicrobial drug sensitivity rates were 73.1%–100.0%. The detection rate of the CRISPR system in 63 strains of *E. coli* extracted from fecal samples from patients with chronic diarrhea was significantly lower than that of 79 strains from healthy people. The detection rate of CRISPR system in 43 high-virulence strains (group B2 and group D) was significantly greater than that in 99 low-virulence strains (group an and B1). The results showed that the fecal isolates of *E. coli* remained highly susceptible to frequently used antibiotics. The CRISPR system may play a significant role in the spread of virulence and drug resistance genes in *E. coli* isolated from feces.

Fan used CRISPR/Cas9 gene editing technology to inhibit the expression of O157:H7Stx gene in enterohemorrhagic *E. coli* (EHEC) and assessed its effect on bacterial growth and cytotoxicity. Fan Huan designed primers for the EHEC O157:H7Stx gene, constructed a CRISPR/Cas9 expression plasmid pdCas9-Stx, and transformed it into EHEC O157:H7 competent cells. RT-PCR and colloidal gold methods were utilized for detect the expression of the Stx gene, and the strain growth curve was drawn. The culture supernatant of the strain was inoculated with Vero cells to observe the cytoplastic condition. The results showed that the successfully constructed pdCas9-Stx expression plasmid could specifically inhibit EHEC O157:H7Stx gene expression and reduce cytotoxicity (Fan et al., 2016).

A toll-like receptor 5 (TLR5) is an influential member of the toll-like receptor family and plays an important regulatory role in the inflammatory response caused by Gram-negative bacteria. Xu knocked out the TLR5 gene of porcine alveolar macrophages (PAMS) by CRISPR/Cas9 gene knockout technology and then counted the colonies to discover the adhesion ability of *E. coli* F18ab and F18ac and *Salmonella* after TLR5 gene knockout (Xu et al., 2021). The results showed that the two designed sgRNAs were successfully inserted into the CRISPR/Cas9 vector with correct sequences, and they could be stably expressed after transfection into porcine alveolar macrophages, and the PCR sequencing peaks showed nested peaks, indicating that the knockout vector had cutting activity. Xu finally obtained a monoclonal cell line TLR5-sg1 with a deletion of 15 bases using public limiting dilution method. The results showed that the transcription level of TLR5 gene was significantly downregulated, and its protein level was also significantly reduced. The results of bacterial adhesion test showed that the number of adherent *E. coli* F18ab, F18ac, and *Salmonella* in TLR5 knockout cells was significantly lower than those in the control group. This study successfully established the porcine TLR5 gene knockout 3D4/21 cell line, and the knockout of TLR5 gene would reduce the adhesion of *E. coli* and *Salmonella* to 3D4/21 cells, which is to further explore the biological function of TLR5 gene. A cell model was established to provide experimental material for in-depth study of the immune regulation role of TLR5 gene in inflammatory response.

## 3 Discussion

Since the discovery of antibiotics, the game between humans and drug-resistant bacteria has never stopped (Hutchings et al., 2019). Antibiotics are widely used in the prevention and treatment of bacterial infections in healthcare system, food production, animal husbandry, and agriculture, bringing convenience to human beings (Manyi-Loh et al., 2018; Hutchings et al., 2019; Roth et al., 2019; Tyrrell et al., 2019). However, the abuse of antibiotics contributed to the occurrence of antibiotic-resistant strains and accelerated the evolution of antibiotic resistance (Laxminarayan et al., 2016; Nadeem et al., 2020). Horizontal transmission of ESKAPE resistance gene accelerated the prevalence of drug-resistant bacteria worldwide (Das et al., 2022). This grim reality requires real-time and rapid diagnosis methods and efficient and specific treatment methods. Since the discovery of CRISPR repeats in bacteria, CRISPR-Cas system has been widely explored in the field of genome editing from being initially considered unimportant outside the field of microbiology. CRISPR-Cas technology has become a milestone discovery in the field of genetic engineering, which has created a brand-new research method for the research in the fields of cell biology and molecular biology (Doudna and Charpentier, 2014; Wang and Doudna, 2023). The application of CRISPR-Cas in fields other than genetic engineering has also developed rapidly, especially in biosensing and infectious disease treatment, which has aroused great interest (Wu et al., 2021; Kostyusheva et al., 2022). In this review, we outline the potential application of CRISPR-Cas technology in ESKAPE, including the development of rapid and instant diagnosis methods and treatments to solve drug-resistant bacterial infections.

Diagnosis based on CRISPR-Cas has developed from an experimental nucleic acid sensing tool to a diagnostic technique for rapid, economical, and ultra-sensitive detection of clinical pathogens. CRISPR-Cas diagnosis is rapidly entering clinical application. For example, the first CRISPR-Cas diagnostic system for SARS-CoV-2-”SHERLOCK”-has been authorized by FDA for emergency use (Gupta et al., 2021). The widespread distribution of “SHERLOCK” enables clinicians and disease control departments to identify pathogens without passing a large number of complex and time-consuming ultra-sensitive tests, which not only reduces the cost of field deployment but also provides opportunities for better controlling the outbreak of infectious diseases (Mustafa and Makhawi, 2021). However, the diagnostic method of CRISPR-Cas is still in the development stage, and many obstacles may still hinder its further development. This technology must overcome many challenges before it can become an exciting new pathogen monitoring method. At present, the main disadvantage of common CRISPR-Cas diagnostic methods is that the nucleic acid of the target pathogen needs to be pre-amplified before detection to achieve sensitivity below the fmol range (Xu et al., 2020). The pre-amplification process not only increases the detection cost but also prolongs the reaction time, and also increases the complexity of the detection process. Modifying Cas protein or crRNA, detecting specific protein markers on the surface of pathogens, and adopting non-primer signal amplification strategy may be potential improvement schemes. Another major drawback is the off-target effect, which will lead to misunderstanding of the results, including false positives. Using bioinformatics methods to carefully design and select the guide RNA with the minimum potential off-target effect and to develop new Cas proteins with the least tolerance to mismatched sgRNA sequences can reduce the unexpected impact of off-target effect (Lee et al., 2016; Li et al., 2018; Li et al., 2019b). A fatal weakness of various diagnostic methods based on CRISPR-Cas is that they cannot quantify the pathogen load and can only provide relatively positive or negative results (van Dongen et al., 2020). How to standardize the results of different patients is another potential bottleneck of CRISPR-Cas diagnosis, which needs complete innovation of detection methods. In addition, the accurate information of genomes of newly emerging or clinically uncommon pathogens has not been fully explored. It is difficult to detect such pathogens on the CRISPR-Cas diagnostic platform, and our failure to retrieve relevant reports on the application of CRISPR-Cas in *E. faecalis* and *E. faecalis* proves this again.

CRISPR-Cas system has become the most efficient and convenient gene editing tool found so far. CRISPR-Cas gene editing technology has broad application prospects, especially in the treatment of many diseases caused by gene mutation or pathogen infection (Strich and Chertow, 2019; Sharma et al., 2021; Katti et al., 2022; Yeh et al., 2022). At present, the common methods of treating drug-resistant bacteria by CRISPR-Cas therapy can be divided into two categories. Targeting specific regions of bacterial chromosomes is a pathogen-centered method, while targeting plasmids carrying drug-resistant genes is another gene-centered method (Shabbir et al., 2019; Ekwebelem et al., 2021). According to different infection situations, a reasonable targeting scheme can eliminate specific strains and reduce the abundance of drug-resistant strains or genes in the host microbial community. The development of new CRISPR-Cas technology will achieve unprecedented control to eliminate drug-resistant bacteria without damaging beneficial bacteria. In addition, we suggest that the treatment of ESKAPE-resistant bacteria may be realized in the following two ways in the future: knocking down the essential host promoter of drug-resistant bacteria during infection, and enhancing the expression of host restriction factors by CRISPRa to improve the host’s resistance to drug-resistant bacteria. Although CRISPR-Cas system has shown impeccable superiority in the treatment of drug-resistant bacteria, it is still only the first step in the clinical treatment of drug-resistant bacteria with CRISPR-Cas. This therapy faces various challenges in terms of safety, delivery, efficiency, and regulatory approval. The first is the most important safety in clinical treatment, which is a matter of priority before any kind of therapy is widely used. The off-target effect of CRISPR-Cas leads to nonspecific cleavage and cytotoxicity (Lin et al., 2014; Ortinski et al., 2017). The off-target effect can be predicted by various computer design tools, such as DeepCRISPR and CRISPRitz. Some strategies have been adopted to reduce the mis-effect (Chuai et al., 2018; Cancellieri et al., 2020). It should also be noted that the natural inhibitor of CRISPR-Cas system—the anti-CRISPR-Cas system—has been widely used to minimize off-target effect and improve target specificity (Aschenbrenner et al., 2020; Dolgin, 2020). The lack of efficient *in vivo* delivery system is another major bottleneck of the CRISPR-Cas system. So far, several potential strategies for transient *in vivo* expression of CRISPR-Cas components have been introduced, including viruses, plasmids, lipid nanoparticles, and extracellular vesicles (Elaswad et al., 2018; Campbell et al., 2019; Chen et al., 2019). Considering that it may be more difficult for the CRISPR-Cas system to enter bacteria than host cells, it is necessary to find safer delivery and powerful gene editing methods to apply it to ESKAPE therapy.

The CRISPR-Cas system is a powerful tool for gene editing. There are still many discussions about CRISPR-Cas technology, especially on the molecular level to fight against several pathogens that seriously endanger patients’ lives and health. Although CRISPR-Cas technology has outstanding advantages in the diagnosis and treatment of ESKAPE infection, more research is still in the laboratory stage, and there is still a long way to go from emerging technology to commercialization. Although it is still in its infancy and even needs a new biotechnology revolution to achieve a technological leap, the diagnosis and treatment methods based on CRISPR-Cas technology are the dominant players of the potential rules of the game. Using the method based on CRISPR-Cas to diagnose and treat pathogens including ESKAPE is a new reality in the field of clinical response to drug-resistant bacteria. In a word, solving the challenges and obstacles including effectiveness, specificity, and safety, and releasing the full potential of CRISPR-Cas system will bring exciting hope for preventing and fighting antibiotic resistance.

## Author contributions

YQ and DZ performed literature search, led the review of the literature, and were involved in the visualization of concepts and in writing the first draft. ML provided revisions and additional conceptual input to the manuscript. YZ and HL prepared the figures. LY and ZY provided assistance for data acquisition. GH conceived, supervised, and finalized the work for submission. All authors contributed to the article and approved the submitted version.
